# High-Quality Draft Genome Sequence of Pseudomonas wadenswilerensis CCOS 864^T^

**DOI:** 10.1128/MRA.01059-18

**Published:** 2018-10-25

**Authors:** Dominik Rutz, David Frasson, Martin Sievers, Jochen Blom, Fabio Rezzonico, Joël F. Pothier, Theo H. M. Smits

**Affiliations:** a Environmental Genomics and Systems Biology Research Group, Institute of Natural Resource Sciences, Zurich University of Applied Sciences (ZHAW), Wädenswil, Switzerland; b Microbiology and Molecular Biology Research Group, Institute of Chemistry and Biotechnology, Zurich University of Applied Sciences (ZHAW), Wädenswil, Switzerland; c Bioinformatics and Systems Biology, Justus-Liebig-Universität, Giessen, Germany; Queens College

## Abstract

Pseudomonas wadenswilerensis CCOS 864^T^ was isolated in 2014 from forest soil. The organism belongs taxonomically to the Pseudomonas putida group, members of which have been well studied for their potential in biotechnological applications. We present here the draft genome sequence of P. wadenswilerensis CCOS 864^T^.

## Abstract (precis)

Pseudomonas wadenswilerensis CCOS 864^T^ was isolated in 2014 from forest soil. The organism belongs taxonomically to the Pseudomonas putida group, members of which have been well studied for their potential in biotechnological applications.

## ANNOUNCEMENT

The genus *Pseudomonas* belongs to the gamma subclass of *Proteobacteria* and forms a large taxonomic grouping of species (1, 2). Members of this genus appear as Gram negative and are involved in a highly diverse range of activities (2). Pseudomonads can be isolated from many different environmental sources, including soil, water, plants, animals, and air (3). Over the years, members of the Pseudomonas putida group were studied because of their usage as an efficient cell factory in industrial biotechnological applications (4). Nowadays, *Pseudomonas* spp. are used for the production of bio-based polymers, small-molecular-weight chiral compounds, heterologous proteins, biosurfactants, and several biotechnologically important enzymes (lipases, proteases, and amylases) (3–5). In 2014, a new species of *Pseudomonas* was isolated during the search for novel biocatalysts. The isolate, identified as Pseudomonas wadenswilerensis CCOS 864^T^, was obtained from soil samples collected in the Riedholz Forest in Richterswil, Switzerland (6).

Genomic DNA of P. wadenswilerensis CCOS 864^T^, grown overnight at 28°C in LB medium, was isolated using the NucleoSpin tissue kit (Macherey-Nagel, Düren, Germany) and fragmented using the Covaris E220 ultrasonicator (average target size, 550 bp). Library preparation was done using the Illumina NeoPrep library system according to the manufacturer’s instructions. Genome sequencing was done at Zurich University of Applied Sciences (ZHAW) using an Illumina MiSeq instrument (2 × 300-bp reads) with a total output of 1,960,890 reads (6). For *de novo* assembly, the SeqMan NGen software version 12.1.0 (DNASTAR, Madison, WI) with standard settings was used. After several assembly steps, a final 18 contigs and an *N*_50_ value of 532,738 bp were obtained. The final assembly had a total length of 5,966,942 bp and a G+C content of 63.39%. After annotation in GenDB (7), comparative genomics to related pseudomonads was performed in EDGAR version 2.3 (8).

By determination of the genome-to-genome distances values (GGDC) based on *in silico* DNA-DNA hybridization (DDH) (9) and calculation of average nucleotide identities (ANIb) with JSpeciesWS version 3.0.20 (10), we confirmed the species delineation against other species of the P. putida group. An average ANIb result of 92.39% ± 0.03% and GGDC of 51.2% ± 0.1% DDH were obtained with three genomes from the species Pseudomonas donghuensis (11, 12), while all other members of the P. putida group had lower ANIb and GGDC values. Therefore, the genome sequence confirms that P. wadenswilerensis CCOS 864^T^ constitutes a separate species distinguishable from other members of the P. putida group. The analysis of the genome content will still show the unique properties of this strain.

### Data availability.

The draft genome sequence of P. wadenswilerensis CCOS 864^T^ has been deposited in DDBJ/EMBL/GenBank under the BioProject number PRJEB27830 and the sequence accession number UIDD00000000. The version described in this paper is version UIDD01000000. Raw sequence reads (Illumina) have been deposited in the Sequence Read Archive under the accession number ERR2814815.

## References

[1] Palleroni NJ 2015 Genus I. *Pseudomonas*, p 323–379. *In* Brenner DJ, Krieg NR, Staley JT, Garrity GM (ed), Bergey’s manual of systematics of archaea and bacteria. John Wiley & Sons, Chichester, United Kingdom.

[2] Timmis KN 2002 *Pseudomonas putida*: a cosmopolitan opportunist *par excellence*. Environ Microbiol 4:779–781. doi:10.1046/j.1462-2920.2002.00365.x. 12534460

[3] Peix A, Ramírez-Bahena M-H, Velázquez E 2018 The current status on the taxonomy of *Pseudomonas* revisited: an update. Infect Genet Evol 57:106–116. doi:10.1016/j.meegid.2017.10.026. 29104095

[4] Poblete-Castro I, Becker J, Dohnt K, dos Santos VM, Wittmann C 2012 Industrial biotechnology of *Pseudomonas putida* and related species. Appl Microbiol Biotechnol 93:2279–2290. doi:10.1007/s00253-012-3928-0. 22350258

[5] Kahlon RS 2016 *Pseudomonas* for industrial biotechnology, p 282–327. *In* Kahlon RS (ed), *Pseudomonas*: molecular and applied biology. Springer, New York, NY.

[6] Frasson D, Opoku M, Picozzi T, Torossi T, Balada S, Smits THM, Hilber U 2017 *Pseudomonas wadenswilerensis* sp. nov. and *Pseudomonas reidholzensis* sp. nov., two novel species within the *Pseudomonas putida* group isolated from forest soil. Int J Syst Evol Microbiol 67:2853–2861. doi:10.1099/ijsem.0.002035. 28820109

[7] Meyer F, Goesmann A, McHardy A, Bartels D, Bekel T, Clausen J, Kalinowski J, Linke B, Rupp O, Giegerich R, Pühler A 2003 GenDB—an open source genome annotation system for prokaryote genomes. Nucleic Acids Res 31:2187–2195. doi:10.1093/nar/gkg312. 12682369 PMC153740

[8] Blom J, Kreis J, Spänig S, Juhre T, Bertelli C, Ernst C, Goesmann A 2016 EDGAR 2.0: an enhanced software platform for comparative gene content analyses. Nucleic Acids Res 44:W22–W28. doi:10.1093/nar/gkw255. 27098043 PMC4987874

[9] Meier-Kolthoff JP, Auch AF, Klenk H-P, Göker M 2013 Genome sequence-based species delimitation with confidence intervals and improved distance functions. BMC Bioinformatics 14:60. doi:10.1186/1471-2105-14-60. 23432962 PMC3665452

[10] Richter M, Rosselló-Móra R, Oliver Glöckner F, Peplies J 2016 JSpeciesWS: a Web server for prokaryotic species circumscription based on pairwise genome comparison. Bioinformatics 32:929–931. doi:10.1093/bioinformatics/btv681. 26576653 PMC5939971

[11] Gao J, Xie G, Peng F, Xie Z 2015 *Pseudomonas donghuensis* sp. nov., exhibiting high-yields of siderophore. Antonie Van Leeuwenhoek 107:83–94. doi:10.1007/s10482-014-0306-1. 25331337

[12] Krzyzanowska DM, Ossowicki A, Jafra S 2014 Genome sequence of *Pseudomonas* sp. strain P482, a tomato rhizosphere isolate with broad-spectrum antimicrobial activity. Genome Announc 2:e00394-14. doi:10.1128/genomeA.00394-14. 24970823 PMC4073107

